# Applying the Hierarchy of Controls: What Occupational Safety Can Teach us About Safely Navigating the Next Phase of the Global COVID-19 Pandemic

**DOI:** 10.3389/fpubh.2021.747894

**Published:** 2021-11-05

**Authors:** Neil J. Sehgal, Donald K. Milton

**Affiliations:** ^1^Department of Health Policy and Management, University of Maryland School of Public Health, College Park, MD, United States; ^2^Maryland Institute for Applied Environmental Health, University of Maryland School of Public Health, College Park, MD, United States

**Keywords:** COVID-19, SARS-CoV-2, transmission, hierarchy of controls, layered defenses, layered prevention, PPE

## Abstract

Eighteen months into the COVID-19 pandemic, and as the world struggles with global vaccine equity, emerging variants, and the reality that eradication is years away at soonest, we add to notion of “layered defenses” proposing a conceptual model for better understanding the differential applicability and effectiveness of precautions against SARS-CoV-2 transmission. The prevailing adaptation of Reason's Swiss cheese model conceives of all defensive layers as equally protective, when in reality some are more effective than others. Adapting the hierarchy of controls framework from occupational safety provides a better framework for understanding the relative benefit of different hazard control strategies to minimize the spread of SARS-CoV-2.

## Introduction

The recent popularity (1) of the application of James Reason's Swiss cheese model of accident causation to COVID-19 transmission falls short in one important regard—the successive “layers” of defense are too easily perceived as equally effective to control the hazard. A preponderance of evidence now demonstrates that this is not the case (2, 3). Inadequacy in defenses and differential risk (e.g., fabric face coverings as compared to filtering respirators, and aerosol “super-spreading” as compared to droplet or fomite transmission) requires a different approach to conceptualizing COVID-19 risk reduction—particularly as COVID-19 continues to devastate the developing world, new and more transmissible variants emerge, vaccines are not yet equitably available across the globe, and eradication of SARS-CoV-2 is decreasingly likely (4). Additionally, framing defensive layers as equally effective may poses challenges to the adoption of more effective mitigation strategies (e.g., vaccination), when the adoption of seemingly equivalent protections may be preferable (5).

## The Hierarchy of Controls

**Figure 1:**
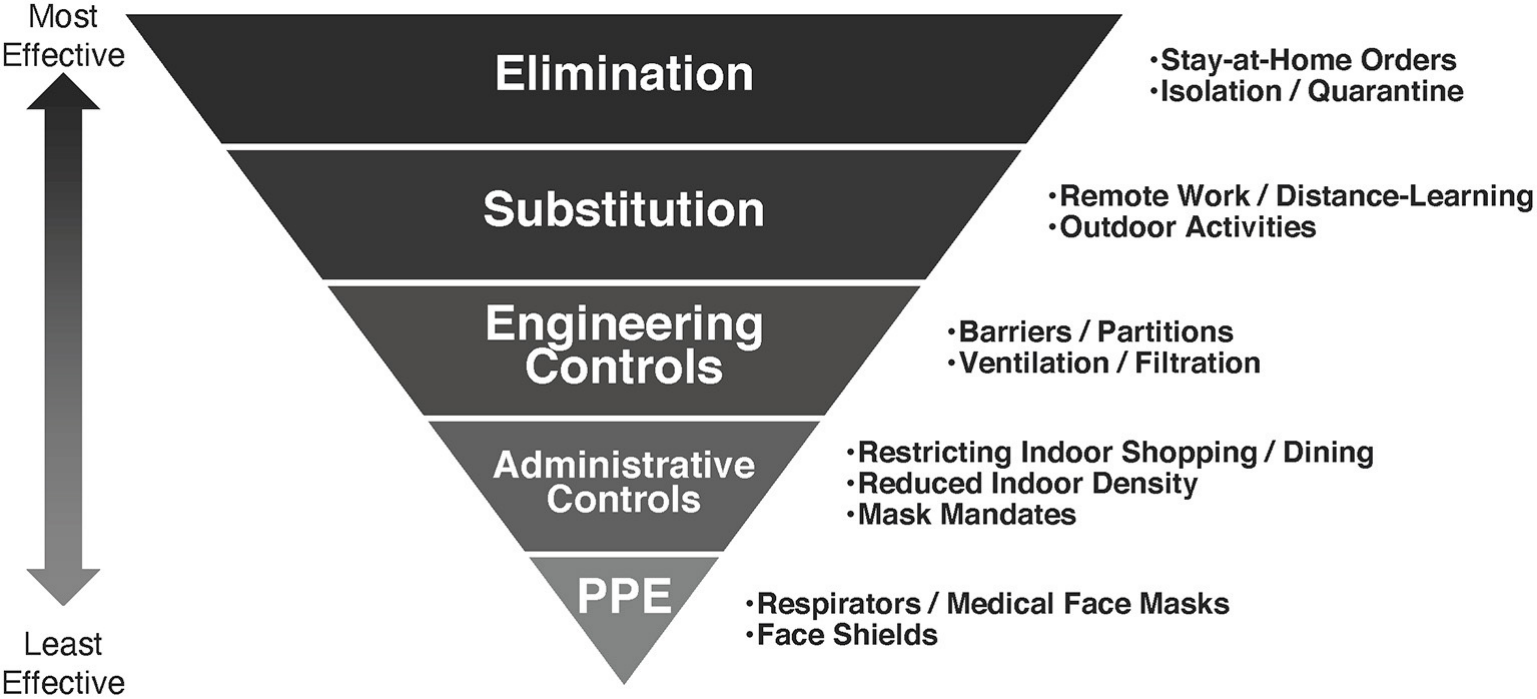
The hierarchy of COVID-19 controls.

The hierarchy of controls is a framework employed in occupational safety and health to better understand the relative effectiveness of different strategies for risk reduction, and to help determine how to implement feasible and effective solutions (6). The model (Figure 1) is represented as an upside-down pyramid, with five categories represented in descending order of effectiveness: elimination, substitution, engineering controls, administrative controls, and personal protective equipment (PPE). And, while developed to better manage exposures to occupational hazards and protect workers, the model has broad applicability in helping healthcare workers, policy makers, and the public better understand the relative effectiveness of strategies to prevent the transmission of an airborne infectious virus like SARS-CoV-2, and the paradigm is increasingly being adopted to conceptualize COVID-19 risk reduction (7, 8). The fundamental idea behind the hierarchy is that, while different hazard controls are effective at minimizing risk, those at the top of the model are more protective than those at the bottom. As in occupational safety and health, employing the most effective methods first and most frequently can best minimize the risk of COVID-19 transmission.

Recent revisions to Centers for Disease Control and Prevention guidance on transmission notes “infections with respiratory viruses are principally transmitted through three modes: contact, droplet, and airborne.” (9) Contact or “fomite” transmission occurs through touching an infectious person or a surface or item that is contaminated with virus. Droplet transmission occurs through exposure to virus-containing respiratory droplets directly from an infectious person to a susceptible person at close range. Airborne transmission occurs through exposure to smaller virus-containing droplets and particles that float in the air, are highly concentrated close to the person who exhaled them and can remain suspended in air for many seconds to hours and accumulate in the air of poorly ventilated spaces. A key weakness in early COVID-19 response was in treating these hazards as equivalent; recent research has demonstrated comparatively low risk of contact transmission (10), and the World Health Organization has noted that despite evidence of the survival of SARS-CoV-2 on certain surfaces, no reports have directly demonstrated fomite transmission (11). Emerging evidence, on the other hand, suggests significant risk associated with close-contact transmission over short distances and in poorly ventilated spaces, with airborne transmission making the largest contribution (12, 13). Though protecting against each of these hazards is necessary, currently employed controls are not equally effective against each, and higher-order controls should be prioritized.

### Elimination and Substitution

Elimination and substitution, the most effective hazard controls, involve physically removing the hazard and associated risk or substituting the hazard with something less risky. Stay-at-home orders employed early in the pandemic were attempts to eliminate transmission risk in communities. While in principle effective, the dire social, psychological, and economic impacts of “lock-downs” limits their long-term viability and necessitate that they be used only briefly while other measures are put in place. Isolation and quarantine are additional means of eliminating transmission risk from infected or potentially infectious individuals. As a precautionary measure, eliminating unnecessary public outings or gatherings is another effective means of removing the risk of SARS-CoV-2 infection. Similarly, allowing remote work or moving indoor-activities outdoors substitutes the hazards incumbent in in-person activities with less risky alternatives. Vaccines against COVID-19 have proven to be a very effective pharmacologic elimination strategy where available.

### Engineering Controls

Engineering controls do not eliminate hazards, but rather isolate individuals from them. Physical barriers separating individuals who must interact at close range are a now very common example of an engineering control. Importantly, however, as they do not eliminate hazards engineering controls must be employed in concert with other controls. Improving the safety of indoor air by increasing ventilation and filtration or employing upper-room germicidal ultraviolet light are as-yet underemployed engineering controls which can further minimize COVID-19 transmission risk (14, 15). Well-designed engineering controls can be highly effective, reducing risk to individuals independent of their own behaviors, and can significantly enhance protection for individuals adherent to administrative controls or employing PPE (16). A key limitation of engineering controls as a community mitigation strategy is that, unlike in industry, it is not always possible to redesign indoor spaces, ventilation systems, and other infrastructure to sufficiently ameliorate the risk of pathogenic transmission. Where engineering controls are able to limit concentrations of indoor respiratory aerosols, however, they are effective in reducing “far-field” airborne transmission of infectious agents like SARS-CoV-2.

### Administrative Controls

Administrative controls involve changing individual behaviors via policy or mandate to minimize hazard risk, and are the control most frequently instituted to increase social distancing, and to reduce person-to-person interaction or population density in defined spaces to protect against COVID-19 infection. Policies restricting indoor activities like large gatherings or dining, and organizational-level mandates like remote-work, staggered in-person work schedules, or distance learning are administrative means of minimizing density-attributed risk. Vaccine and mask-mandates are another administrative control, though their effectiveness has been limited by social and political resistance. This illustrates the key weakness of reliance on administrative controls (and the challenge in relying exclusively on them to mitigate transmission risk); compliance is necessary for administrative controls to be effective, and even well-intentioned individuals are prone to slips and lapses in adherence. And, as lessons from patient safety and risk management teach, policies that rely on perfect adherence are inadequate and doomed to fail.

### Personal Protective Equipment

The final risk reduction strategy is the employment of PPE, protecting individuals from known hazard exposure using respirators, eye protection, and other individually donned protective items. PPE can reduce risk of hazard and its use has been an essential strategy to limit COVID-19 transmission, though it is necessarily less protective than controls higher in the hierarchy. The effectiveness of PPE as a control is additionally limited as it is reliant on both adequate supply and proper and continuous use. In circumstances where hazards are truly unavoidable, PPE use is critical. In work-settings reliant on PPE for hazard control, regulations mandate additional controls as well. Lay-persons are infrequently trained in the correct use of PPE, and in the current pandemic frequently ill-equipped—non-medical masks and fabric face coverings are not PPE, and wearing a non-medical mask does not eliminate SARS-CoV-2 infection risk to the individual (17).

## Discussion

As it is increasingly likely that COVID-19 will remain endemic, as more transmissible variants emerge, and as at our current pace we may not vaccinate people in low-income countries until the end of 2022 or beyond (18), our continued reliance on the least effective controls and our continued treatment of controls as substitutes instead of complements—such as reliance on face-coverings or social distancing, but not both—has limited our ability to keep SARS-CoV2 in-check. The hierarchy of controls is an effective model for understanding both the relative effectiveness of hazard minimization, and that while most strategies are necessary few are sufficient to slow the spread of COVID-19.

## Data Availability Statement

The original contributions presented in the study are included in the article/supplementary material, further inquiries can be directed to the corresponding author.

## Author Contributions

NS conceptualized the piece and drafted the first version of the manuscript. NS and DM commented on and edited the draft. Both authors reviewed and agreed with the final version.

## Conflict of Interest

The authors declare that the research was conducted in the absence of any commercial or financial relationships that could be construed as a potential conflict of interest.

## Publisher's Note

All claims expressed in this article are solely those of the authors and do not necessarily represent those of their affiliated organizations, or those of the publisher, the editors and the reviewers. Any product that may be evaluated in this article, or claim that may be made by its manufacturer, is not guaranteed or endorsed by the publisher.
